# Whole-exome sequencing reveals a novel CHM gene mutation in a family with choroideremia initially diagnosed as retinitis pigmentosa

**DOI:** 10.1186/s12886-015-0081-4

**Published:** 2015-07-28

**Authors:** Hui Guo, Jisheng Li, Fei Gao, Jiangxia Li, Xinyi Wu, Qiji Liu

**Affiliations:** Department of Ophthalmology, Qilu Hospital of Shandong University, 107#, Wenhua Xi Road, Jinan, 250012 China; Department of Medical Oncology, Cancer Center, Qilu Hospital of Shandong University, Jinan, China; Department of Medical Genetics, Key Laboratory for Experimental Teratology of the Ministry of Education, Shandong University School of Medicine, 44#, Wenhua Xi Road, Jinan, 250012 China

**Keywords:** CHM, Choroideremia, Mutation, Retinitis pigmentosa, Whole-exome sequencing

## Abstract

**Background:**

Genomic mutations in about 200 genes are associated with hereditary retinal diseases. In this study, we screened for the disease-causing gene mutation in a family with X-linked retinal degenerative disease.

**Methods:**

Pedigree data were collected and genomic DNA was isolated from peripheral blood of family members, who also underwent comprehensive ophthalmic examination including visual acuity, slit-lamp examination, fundus examination and visual field testing at Qilu Hospital of Shandong University. Whole-exome genomic sequencing was used to screen for gene mutations in the male proband. Sanger sequencing was used to confirm the mutation revealed in this family.

**Results:**

Two affected males underwent ophthalmic examination; retinitis pigmentosa (RP) was diagnosed on the basis of night blindness beginning at an early age, decreasing visual acuity, progressive loss of peripheral vision, attenuation of retinal vessels and pigment disturbance on fundus examination. However, whole-exome sequencing revealed no mutation in RP-associated genes. Instead, we identified a novel hemizygous c.1475_1476insCA mutation in the choroideremia-associated gene (*CHM*). The mutation was confirmed by Sanger sequencing and further excluded from the possibility as a rare polymorphism. From the genetic data and clinical findings, the diagnosis was corrected to choroideremia (CHM). Further molecular genetic analysis suggested that this novel *CHM* mutation caused a frame shift (p.Leu492PhefsX7) and encoded a truncated nonfunctional Rab escort protein 1 (REP-1), which caused CHM in this family. Finally, sequencing data for a pregnant female member confirmed that she did not carry the mutation and thus was carrying a healthy infant.

**Conclusion:**

We report a novel *CHM* mutation, c.1475_1476insCA, identified by whole-exome sequencing in a family with X-linked CHM initially diagnosed as RP. Our findings emphasize the value of a diagnostic approach that associates genetic and ophthalmologic data to facilitate the proper clinical diagnosis of rare hereditary retinal diseases such as CHM.

## Background

Blindness is one of the most-feared maladies in humans. The World Health Organization estimated that in 2009, about 314 million people were visually impaired, among whom about 45 million were blind [1]. Progressive inherited retinal degenerative diseases, including age-related macular degeneration and retinitis pigmentosa (RP), as well as the rare choroideremia (CHM), are the leading causes of blindness in developed countries, affecting about one-third of all people older than 75 [2].

RP is the most common retinal hereditary disease and refers to various forms of progressive retinal degeneration with predominantly impaired rod photoreceptors [3]. It is a clinically and genetically highly heterogeneous retinal disease and is characterized by different genetic transmission modes including autosomal dominant, autosomal recessive and X-linked [3, 4]. Mutations in 62 genes are associated with RP [4–6]. In contrast, CHM is a rare X-linked progressive-inherited retinal degenerative disease characterized by progressive degeneration of the choriocapillaris, retinal pigment epithelium and photoreceptors [7]. The term choroideremia refers to the absence (−eremia) of the choroid. The incidence of CHM ranges from 1:50,000 to 1:100,000 [8, 9]. CHM is caused by mutations in Rab escort protein 1 (*REP-1*), which encodes a protein involved in vesicular trafficking [9–11]. Males with CHM exhibit progressive vision loss at a young age, usually beginning with night blindness and sometimes progressing to complete blindness later in life; female carriers are generally asymptomatic. However, a heterozygous female may occasionally show mild symptoms [12, 13].

CHM and RP share several common clinical features including night blindness, constriction of the visual field, gradually reduced visual acuity, and retinal degeneration, which may lead to difficulties in the differential diagnosis and even cause diagnostic confusion, especially with the absence of a typical fundus appearance [14].

We used whole-exome sequencing to screen for the disease-causing gene mutation for the male proband in a family with X-linked retinal degenerative disease initially diagnosed as RP. We found a novel mutation in *CHM* in the family that was further confirmed by Sanger sequencing and excluded from the possibility as a rare gene polymorphism. No mutation was revealed in RP-associated genes. Combining the genetic data and clinical findings, the diagnosis was corrected to choroideremia for this family. The identified novel c.1475_1476insCA mutation in *CHM* caused a frame shift (p.Leu492PhefsX7), and the mutant gene encoded a 497 amino acid truncated nonfunctional REP-1 protein. Our findings emphasize the value of a diagnostic approach that associates genetic and ophthalmologic data to facilitate the proper clinical diagnosis for rare hereditary retinal diseases such as CHM.

## Methods

### Participants and clinical data

A family with X-linked hereditary retinal degenerative disease, with 4 affected male members, was recruited in the Department of Ophthalmology, Qilu Hospital of Shandong University. The proband and another affected male underwent full ophthalmic examination, including visual acuity, slit-lamp, fundus and visual field examination. Physical examination was performed to exclude systemic diseases. This study was approved by the Medical Ethics Review Board at Qilu Hospital of Shandong University, and following the principles of the Declaration of Helsinki, informed consent was obtained from all subjects before entry into this study. Peripheral venous blood samples were collected from the 2 affected males and a female carrier for genomic DNA extraction from leucocytes using standard protocols.

### Whole-exome sequencing

Samples for the male proband underwent whole-exome sequencing by BGI Shenzhen (Beijing Genome Institute, Shenzhen, China). Briefly, the qualified genomic DNA sample was randomly fragmented by Covaris and the size of the library fragments was mainly distributed between 150 to 200 bp. Then adapters were ligated to both ends of the resulting fragments. The adapter-ligated templates were purified by the Agencourt AMPure SPRI beads and fragments with insert size about 250 bp were excised. Extracted DNA was amplified by ligation-mediated PCR (LM-PCR), purified, and hybridized to the SureSelect Biotiny lated RNA Library (BAITS) (Agilent, Santa Clara, CA, USA) for enrichment, hybridized fragments were bound to the strepavidin beads whereas non-hybridized fragments were washed out after 24 h. Captured LM-PCR products were subjected to Agilent 2100 Bioanalyzer to estimate the magnitude of enrichment. Each captured library was then loaded on Hiseq2000 platform (Illumina, San Diego, CA, USA), and we performed high-throughput sequencing for each captured library to ensure that each sample meets the desired average sequencing depth. Raw image files were processed by Illumina basecalling Software 1.7 for base-calling with default parameters and the sequences of each individual were generated as 90 bp pair-end reads. An 8.5-Gb sequence was generated with at least 98.7 % coverage for 4× and 95.4 % for 10× of the sample. All variations were filtered using dbSNP137, the 1000 Genomes Project, and HapMap8 databases. Coverage of target region is 99.4 %. Data were reviewed for all genes known to be associated with hereditary retinal disease.

### Sanger sequencing

Sanger sequencing was used to confirm the mutation in *CHM* gene detected by whole-exome sequencing. The sequence containing the mutation found was amplified by PCR with the primer pairs CHMF, 5′ AGAGGTGTTTGGGATTTC3′, and CHMR, 5′ TAGGTAAGGGGATGGTGT 3′. Variants in available family members were also analyzed. Novel variants were then evaluated in 200 healthy controls.

## Results

### Clinical findings

**Fig. 1:**
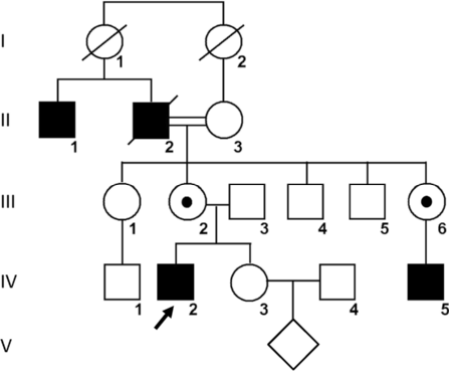
Pedigree of mainland Chinese family with inherited retinal degenerative disease. X-linked inheritance pattern is shown. Closed symbols indicate affected patients and open symbols unaffected subjects. Dotted circles indicate female carriers. Arrow indicates proband. Slash indicates deceased person

The pedigree of this family showed X-linked transmission with 13 living family members, including 3 living affected males and 2 female carriers (Fig. 1). All affected males had experienced poor night vision at an early age and decreased visual acuity. Physical examination of the male proband IV-2 (31 years old) and another affected male IV-5 (25 years old) excluded systemic disorders. The best-corrected visual acuity for IV-2 was 1/20 OD with myopia −1.50 and 2/20 OS with myopia −2.00D; the best-corrected visual acuity for IV-5 was 6/20 OD with myopia −2.25 and 5/20 OS with myopia −3.00D. The anterior segment parameters showed no abnormalities for either.

**Fig. 2:**
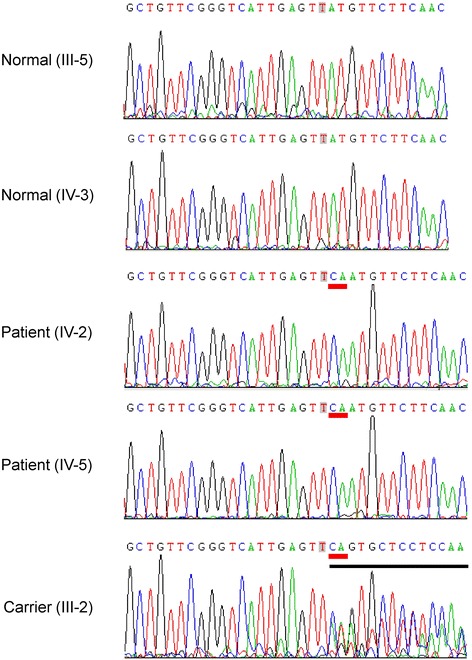
Images of fundus changes in the proband IV-2 and another affected male IV-5. Areas of retinal pigment epithelium (RPE) disrupted, chorioretinal atrophy, loss of choriocapillaris, and scattered bone-spicule pigment deposits partially covering the central macula in IV-2. Less severe chorioretinal atrophy and much less bone-spicule pigment deposits with preservation of the central macula in the younger patient IV-5

**Fig. 3:**
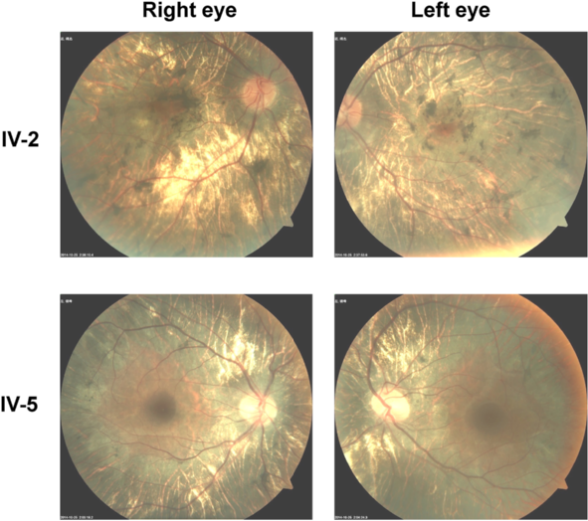
Visual field examination showing severe visual field constriction for both the proband IV-2 and another affected male IV-5

Unlike the normal homogeneous brown background of melanin pigment in the normal retinal pigment epithelium (RPE) and choroid, the retina of the proband IV-2 showed profound chorioretinal atrophy (Fig. 2). The fundus for IV-2 showed areas of RPE disruption, severe chorioretinal atrophy, loss of choriocapillaris, and scattered bone-spicule pigment deposits partially covering the central macula (Fig. 2). Meanwhile, the fundus for IV-5 showed less severe chorioretinal atrophy and much less bone-spicule pigment deposits, with preservation of the central macula, which suggests an earlier disease stage for IV-5 than IV-2. Both eyes of IV-5 and IV-2 showed a tubular visual field, which suggests severe neuropathy (Fig. 3). Thus, the family received a diagnosis of RP based on night blindness beginning at an early age, decreasing visual acuity, progressive loss of peripheral vision, attenuated of retinal vessels and typical bone-spicule pigment deposits on fundus examination.

### Whole-exome sequencing

Whole-exome sequencing generated about 8.5 Gb of sequence data for the tested individual IV-2. 98.7 % of the region was at least 4× sequenced and 95.4 % 10× sequenced. Coverage of target region is 99.4 %. Totally 135843 SNPs and 13952 indel variations were detected in the exome analysis. After quality control, the results reveal no mutation in RP-associated genes for the male proband IV-2. Instead, a novel hemizygous insertion mutation c.1475_1476insCA at the CHM gene location was detected for the proband IV-2 (based on NM_000390.2). Molecular genetic analysis suggested that this insertion mutation caused a frame shift (p.Leu492PhefsX7) and encoded a truncated, 497 amino acid (aa), nonfunctional REP-1 protein.

### Sanger sequencing

**Fig. 4:**
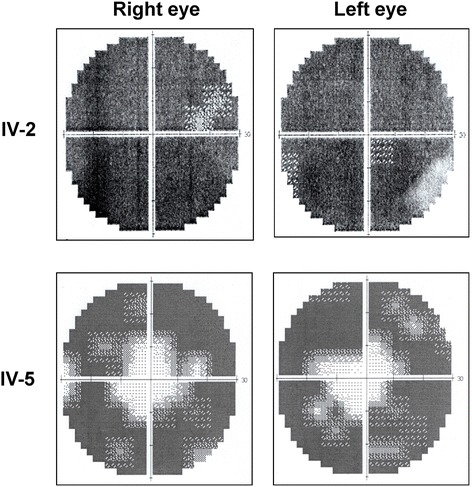
Sanger sequencing chromatography of the c.1475_1476insCA mutation in choroideremia-associated gene (*CHM*). No mutations were found in 2 healthy family members, III-5 and IV-3. Red bar indicates the inserted CA nucleotides in CHM gene of proband IV-2, another affected male IV-5 and a female carrier III-2. Black bar indicates the mixed sequencing behind the insertion site in the hemizygous CHM mutation carrier III-2

Sanger sequencing confirmed the CHM gene c.1475_1476insCA mutation in the affected males IV-2 and IV-5 (Fig. 4). Furthermore, this novel CHM mutation was detected in none of 200 healthy controls, which excluded the mutation as being a rare DNA polymorphism. A female carrier III-2, the mother of the proband IV-2, was heterozygous for the insertion mutation (Fig. 4). In addition, Sanger sequencing data suggest that affected male III-5 did not have the disease-causing CHM gene mutation, which agreed with his disease-free status. Finally, sequencing data for the pregnant female member IV-3 showed that she did not carry the mutation and thus was carrying a healthy infant.

## Discussion

Using whole-exome and Sanger sequencing, we identified a novel hemizygous CHM mutation, c.1475_1476insCA, in a family with retinal degenerative disease initially diagnosed as RP. This novel CHM insertion mutation, rather than being a rare polymorphism in the general population, resulted in a truncated protein, commonly observed in CHM families. By combining the clinical data and initial genetic findings, the diagnosis for disease in this family was suggested to be an atypical form of CHM.

CHM is a rare X-linked retinal degenerative disease caused by mutations in the *CHM* gene that encodes REP-1 [12]. CHM mutations cause loss of functioning REP-1, an essential component of an enzyme complex formed with Rab geranylgeranyltransferase. Without functioning REP-1, RABs cannot participate in pathways of intracellular vesicular transport [12]. REP-1 is normally expressed in humans, and loss of REP-1 protein can be compensated by REP-2 in all tissues, except in the eye [15]. Functioning REP-1 is crucial for normal biological function of the retinal pigment epithelium and photoreceptors. Ultimately, lack of REP-1 results in the degeneration of these cells, as well as associated choroidal tissue [16].

The REP-1 protein-coding gene *CHM* spans 186,383 bp on Xq21.2 (based on NC_000023.11). A wide variety of CHM-causing mutations include small deletions, nonsense mutations, missense mutations, frame shifts, splice site defects, retrotransposon insertions and deletion of the entire CHM gene [17]. At least 147 CHM mutations have been reported in patients with choroideremia [5]. Thus, sequencing of the CHM gene has emerged as a diagnostic tool to identify mutations causing CHM [18]. There are two transcript variants for *CHM* gene. The 5442-bp CHM transcript variant 1 mRNA consists of 15 exons (NM_000390.2) with an open reading frame of 1962 bp and encodes a 653-aa REP-1 protein (95 kDa), while the 2856-bp CHM transcript variant 2 mRNA consists of 5 exons (NM_001145414.2) with an open reading frame of 333 bp and encodes a 110-aa REP-1 isoform protein. The two transcript variants share the same four 5′ exons and the exon 5 of the shorter variant is actually located in the intron 4 of the long transcript. As summarized in the CHM database (http://www.lovd.nl/CHM), no mutation in the exon 5 of the shorter transcript has been reported to cause choroideremia. And about 75 % (209/279) of the diseasing causing *CHM* gene mutations summarized are located in the latter 5 exons of the longer variant (exon 5 to 15). And totally 9 known mutations in exon 12, in which the novel c.1475_1476insCA mutation is located, have been identified to cause choroideremia in literature according to above database.

The c.1475_1476insCA insertion mutation we identified in exon 12 induced a frame shift which caused a new premature stop codon. Subsequently, the 156 C-terminal residues of REP-1 protein were truncated in the encoded mutant protein, leaving only 497 residues of the 653-aa protein. Most of the CHM-causing mutations result in lack of REP-1 due to a premature stop codon and degradation of the inappropriately folded protein or truncated mRNA [19, 20]. Our data demonstrating the truncation in the CHM gene in CHM patients suggest that a truncated REP-1 protein of 497 aa is unable to function as a normal escort protein of Rab proteins *in vivo*. The truncated REP-1 protein is likely degraded enzymatically in vivo in the affected members of this CHM family.

CHM is a rare eye disease with clinical features similar to those of RP. So far, no effective treatment exists for either disease. Transplantation of autologous transduced iris pigment epithelial cells into the subretinal space might help CHM patients [21]. Clinically, CHM and RP share several features common to retinal degenerative disorders, including night blindness, visual field constriction, visual acuity reduction and retinal degeneration, which may lead to difficulties in the differential diagnosis and even cause diagnosis confusion, especially with lack of typical fundus appearance [14]. On fundus examination, CHM is clinically characterized by chorioretinal scalloped atrophy initiated from the mid-peripheral fundus without affecting the macula [5, 7, 8]. However, these typical fundus changes in CHM may not be apparent when the patient visits the physician. Considering the diverse appearance of fundus in RP patients, CHM patients without typical fundus changes may be easily given a diagnosis of RP [5]. Actually, about 6 % of patients with a diagnosis of RP-related disorders have choroideremia [14].

Consistent with the above reports, the typical fundus changes for CHM including chorioretinal scalloped atrophy with preservation of the macula was not found in the proband with CHM mutations in our family. Instead, fundus examination revealed the typical bone-spicule pigment deposits of RP in both the proband and another brother. Thus as mentioned earlier, this family was initially given a diagnosis of RP based on night blindness, decreasing visual acuity, loss of peripheral vision, and typical bone-spicule pigment deposits. Recently, Li et al. reported mutations in the CHM gene in 6 of 157 families with RP by whole-exome sequencing [5]. However, the fundus changes in the 6 probands with CHM mutations were also atypical as compared with those seen in classical RP, and no potential pathogenic mutations in RP-associated genes were found in the 6 families [5]. Similarly, clinical and experimental data for our family suggest an atypical phenotype of CHM. Together with previous reports, our findings indicate that CHM may be misdiagnosed as RP with lack of a typical fundus appearance and the CHM gene should be included as a candidate in genetic studies for atypical RP.

## Conclusions

In conclusion, we used whole-exome sequencing and identified a novel hemizygous *CHM* mutation, c.1475_1476insCA, in a family with retinal degenerative disease initially diagnosed as RP. Combining the genetic and clinical findings led to correction of the diagnosis to atypical choroideremia. The results highlight the emerging role of whole-exome sequencing in the diagnosis of rare genetic diseases. Our findings emphasize the value of a diagnostic approach that associates genetic and ophthalmologic data to facilitate the proper clinical diagnosis of rare hereditary retinal diseases such as CHM.

## References

[1] Prokofyeva E, Wilke R, Lotz G, Troeger E, Strasser T, Zrenner E (2009). An epidemiological approach for the estimation of disease onset in Central Europe in central and peripheral monogenic retinal dystrophies. Graefes Arch Clin Exp Ophthalmol.

[2] Tucker BA, Mullins RF, Stone EM (2014). Stem cells for investigation and treatment of inherited retinal disease. Hum Mol Genet.

[3] Hartong DT, Berson EL, Dryja TP (2006). Retinitis pigmentosa. Lancet.

[4] den Hollander AI, Black A, Bennett J, Cremers FP (2010). Lighting a candle in the dark: advances in genetics and gene therapy of recessive retinal dystrophies. J Clin Invest.

[5] Li S, Guan L, Fang S, Jiang H, Xiao X, Yang J (2014). Exome sequencing reveals CHM mutations in six families with atypical choroideremia initially diagnosed as retinitis pigmentosa. Int J Mol Med.

[6] Xu Y, Guan L, Shen T, Zhang J, Xiao X, Jiang H (2014). Mutations of 60 known causative genes in 157 families with retinitis pigmentosa based on exome sequencing. Hum Genet.

[7] Roberts MF, Fishman GA, Roberts DK, Heckenlively JR, Weleber RG, Anderson RJ (2002). Retrospective, longitudinal, and cross sectional study of visual acuity impairment in choroideraemia. Br J Ophthalmol.

[8] Coussa RG, Traboulsi EI (2012). Choroideremia: a review of general findings and pathogenesis. Ophthalmic Genet.

[9] MacDonald IM, Sereda C, McTaggart K, Mah D (2004). Choroideremia gene testing. Expert Rev Mol Diagn.

[10] Cremers FP, van de Pol DJ, van Kerkhoff LP, Wieringa B, Ropers HH (1990). Cloning of a gene that is rearranged in patients with choroideraemia. Nature.

[11] van den Hurk JA, Schwartz M, van Bokhoven H, van de Pol TJ, Bogerd L, Pinckers AJ (1997). Molecular basis of choroideremia (CHM): mutations involving the Rab escort protein-1 (REP-1) gene. Hum Mutat.

[12] Seabra MC, Brown MS, Goldstein JL (1993). Retinal degeneration in choroideremia: deficiency of rab geranylgeranyl transferase. Science.

[13] Furgoch MJ, Mewes-Ares J, Radziwon A, Macdonald IM (2014). Molecular genetic diagnostic techniques in choroideremia. Mol Vis.

[14] MacDonald IM, Hume S, Chan S, Seabra MC. Choroideremia. In: Pagon RA, Adam MP, Ardinger HH, Wallace SE, Amemiya A, Bean LJH, et al., editors. GeneReviews® [Internet]. Seattle (WA): University of Washington, Seattle; 1993-2015.2003 Feb 21 [Updated 2015 Feb 26].

[15] Seabra MC, Ho YK, Anant JS (1995). Deficient geranylgeranylation of Ram/Rab27 in choroideremia. J Biol Chem.

[16] Tolmachova T, Anders R, Abrink M, Bugeon L, Dallman MJ, Futter CE (2006). Independent degeneration of photoreceptors and retinal pigment epithelium in conditional knockout mouse models of choroideremia. J Clin Invest.

[17] van den Hurk JA, van de Pol DJ, Wissinger B, van Driel MA, Hoefsloot LH, de Wijs IJ (2003). Novel types of mutation in the choroideremia ( CHM) gene: a full-length L1 insertion and an intronic mutation activating a cryptic exon. Hum Genet.

[18] MacDonald IM, Mah DY, Ho YK, Lewis RA, Seabra MC (1998). A practical diagnostic test for choroideremia. Ophthalmology.

[19] Sergeev YV, Smaoui N, Sui R, Stiles D, Gordiyenko N, Strunnikova N (2009). The functional effect of pathogenic mutations in Rab escort protein 1. Mutat Res.

[20] Iino Y, Fujimaki T, Fujiki K, Murakami A (2008). A novel mutation (967-970 + 2)delAAAGGT in the choroideremia gene found in a Japanese family and related clinical findings. Jpn J Ophthalmol.

[21] Abe T, Yoshida M, Yoshioka Y, Wakusawa R, Tokita-Ishikawa Y, Seto H (2007). Iris pigment epithelial cell transplantation for degenerative retinal diseases. Prog Retin Eye Res.

